# Touch Perception Altered by Chronic Pain and by Opioid Blockade^1,2,3^

**DOI:** 10.1523/ENEURO.0138-15.2016

**Published:** 2016-03-10

**Authors:** Laura K. Case, Marta Čeko, John L. Gracely, Emily A. Richards, Håkan Olausson, M. Catherine Bushnell

**Affiliations:** 1 National Center for Complementary and Integrative Health, NIH, Bethesda, Maryland 20892; 2 Department of Clinical and Experimental Medicine, Center for Social and Affective Neuroscience, Linköping University, Linköping, S-581 83 Sweden

**Keywords:** affective touch, C-tactile fibers, chronic pain, fibromyalgia, opioids, somatosensation

## Abstract

Touch plays a significant role in human social behavior and social communication, and its rewarding nature has been suggested to involve opioids. Opioid blockade in monkeys leads to increased solicitation and receipt of grooming, suggesting heightened enjoyment of touch. We sought to study the role of endogenous opioids in perception of affective touch in healthy adults and in patients with fibromyalgia, a chronic pain condition shown to involve reduced opioid receptor availability. The pleasantness of touch has been linked to the activation of C-tactile fibers, which respond maximally to slow gentle touch and correlate with ratings of pleasantness. We administered naloxone to patients and healthy controls to directly observe the consequences of µ-opioid blockade on the perceived pleasantness and intensity of touch. We found that at baseline chronic pain patients showed a blunted distinction between slow and fast brushing for both intensity and pleasantness, suggesting reduced C-tactile touch processing. In addition, we found a differential effect of opioid blockade on touch perception in healthy subjects and pain patients. In healthy individuals, opioid blockade showed a trend toward increased ratings of touch pleasantness, while in chronic pain patients it significantly decreased ratings of touch intensity. Further, in healthy individuals, naloxone-induced increase in touch pleasantness was associated with naloxone-induced decreased preference for slow touch, suggesting a possible effect of opioid levels on processing of C-tactile fiber input. These findings suggest a role for endogenous opioids in touch processing, and provide further evidence for altered opioid functioning in chronic pain patients.

## Significance Statement

C-tactile fibers are normally more activated by slow gentle touch than by fast touch and send a signal to the brain that contributes to the perception of pleasantness. This paper shows that people with the chronic pain condition fibromyalgia perceive less difference between fast and slow gentle touch in terms of its intensity and pleasantness, suggesting reduced C-tactile fiber processing and/or differences in opioid signaling. Our paper is also the first demonstration in humans that opioids affect how touch feels. In healthy individuals, blocking opioid binding tended to increase touch pleasantness, whereas in fibromyalgia patients it decreased perceived intensity. This suggests a role for endogenous opioids in touch perception, and provides new evidence that opioids function differently in chronic pain.

## Introduction

Touch plays a strong role in social communication and bonding. In mammals, activities such as licking, grooming, and sensual caress seem to be intrinsically rewarding. Primates, for instance, appear to spend more time grooming others than is necessary for hygiene (Dunbar, 2010). These bonding-related types of social touch are associated with activation of C-tactile (CT) fibers, a class of unmyelinated C-fibers present in hairy skin, whose strongest firing is elicited by slow-stroking touch (Loken et al., 2009). Testing of two patients with a rare A-beta fiber neuronopathy (a polyneuropathy involving destruction of the cell bodies of neurons; Sterman et al., 1978) but intact C fibers has demonstrated that CT-optimal touch (touch with stimulus parameters that normally elicit the strongest firing of CT fibers) generates a feeling of pleasantness and robust activation of the insular cortex, a region with a relatively high density of opioid receptors (Olausson et al., 2002, 2008; Vogt, 2005; Baumgärtner et al., 2006). In healthy individuals, the firing rate of CT afferents is positively correlated with the reported pleasantness of touch (Loken et al., 2009), suggesting a possible link between the pleasantness of slow, CT-optimal touch and opioid signaling. The endogenous opioid system is believed to underlie the rewarding nature of social relationships and may mediate the pleasantness and reward of CT-related social touch (Panksepp et al., 1980; Dunbar, 2010). We therefore sought to study response to CT touch through use of an opioid-receptor blockade. We also sought to study the role of opioids in the perception of CT touch by studying patients with a chronic pain condition suggested to involve disruption of opioid processing (Harris et al., 2007).

There is evidence in animals that the rewarding nature of social touch involves opioidergic mechanisms. Indeed, there are opioid receptors throughout the brain, and they are especially concentrated in brain areas related to pain and affect (Baumgärtner et al., 2006). In addition, beta-endorphins increase in the cerebrospinal fluid of monkeys after receiving social grooming following a period of social isolation (Keverne et al., 1989). Naloxone blocks opioid signaling by binding to opioid receptors, which reduces the binding of endogenous opioids. Interestingly, such opioid blockade often causes a drop in mood (Mendelson et al., 1978; Schull et al., 1981; Grevert et al., 1983), and in nonhuman primates, leads to increased receipt of grooming. Martel et al. (1995) administered acute doses of naloxone to rhesus monkeys and found that mature females both sought and received more grooming from their companions under naloxone, though they did not increase their grooming of peers. The authors interpreted this behavior as naloxone blocking the positive affect arising from social contact, leading the monkeys to solicit comfort through increased grooming. Alternatively, naloxone might alter the animal’s social-motivational state, increasing the pleasantness and liking of social touch. Similar results have been found in studies of a variety of monkey species showing increased solicitation and receipt of grooming after injection of µ-opioid antagonists (Meller et al., 1980; Fabre-Nys et al., 1982; Schino and Troisi, 1992; Martel et al., 1995; Graves et al., 2002). Furthermore, in talapoin monkeys, opioid blockade increased requests for grooming, as well as time spent grooming peers, whereas opioid administration reduced grooming requests and grooming of peers (Keverne et al., 1989). Increased solicitation of grooming might reflect an altered mood or motivational state consistent with either increased or decreased liking of the grooming. However, the fact that the primates in these studies not only showed increased solicitation (wanting) of grooming but also received grooming for longer periods of time suggests enhanced liking of grooming after opioid receptor blockade.

The involvement of opioids in human appreciation of CT-targeted touch is unknown. In the current study we examined ratings of the pleasantness of slow touch (CT-optimal) and fast touch (CT non-optimal, but still stimulates CT fibers) in a group of participants with fibromyalgia (FM), a chronic pain condition in which opioidergic abnormalities have been shown (Harris et al., 2007), and compared them to ratings of healthy individuals. We predicted that the chronic pain patients would show a reduced preference for CT-optimal touch (slow touch relative to fast touch) and reduced ratings of touch pleasantness overall based on decreased central µ-opioid receptor availability in FM (Harris et al., 2007) and related alterations in other chronic pain conditions (Jones et al., 1994, 2004; Klega et al., 2010). In addition, we administered naloxone to half of the patients and controls and saline to the other half, and compared their ratings of slow and fast brushing before and after the drug injection. Naloxone is an opiate antagonist used clinically to reverse overdose of opiates, such as morphine; it has a high affinity for the μ-opioid receptor and thus blocks the binding of endogenous endorphins (opioid peptides). This property enabled us to study the role of opioids in the perception of the pleasantness and intensity of CT touch. Naloxone binds a proportion of opioid receptors and thus should decrease the binding of endogenous opioids believed to be released by slow, grooming-like touch. We therefore hypothesized that naloxone would reduce preference for slow (CT-optimal) touch in healthy subjects. Because naloxone increases receipt of grooming in monkeys, however, we also predicted that naloxone would alter the overall pleasantness of brushing (regardless of brushing speed) as opioid withdrawal appears to alter the value of social touch (Loseth et al., 2014). Finally, we hypothesized that these effects would be reduced in chronic pain patients with FM due to reduced µ-opioid receptor availability.

## Materials and Methods

### Participants

Participants were 28 healthy controls (25 female) and 24 chronic pain patients diagnosed with FM (23 female). Participants ranged in age from 18 to 64 (Table 1) and all were fluent in English. Patients and controls were matched at the group level for age, sex, and level of education, and did not differ in weight (*t*_(50)_ = 0.34; *p* = 0.74; Table 1) or body mass index (*t*_(50)_ = 1.21; *p* = 0.23; Table 1). Patients did show higher levels of anxiety (*t*_(48)_ = 3.14; *p* = 0.003) and depression (*t*_(48)_ = 4.15; *p* = 0.0001) than controls on the Hospital Anxiety and Depression Scale (HADS; Zigmond and Snaith, 1983; Table 1). However, scores were in the subclinical range (<10). Participants were recruited through ads placed in local newspapers and at the [National Institutes of Health]. Several patients were recruited from local physicians. All subjects were informed about naloxone, including its pharmacological properties, its clinical use, and its possible side effects. Participants provided informed consent in accordance with approval from the [author institution]. Participants were monetarily compensated for their study participation. All FM participants completed the Fibromyalgia Impact Questionnaire (FIQ; Burckhardt et al., 1991). The mean FIQ score of our participants represented a moderate effect of FM on functioning (Bennett et al., 2009) and was comparable to that of similar FM samples (Martinez et al., 1995; Table 1 shows the mean score).

**Table 1.** Participant demographics

	***N***	**Age**	**Sex**	**Weight**	**Anxiety (HADS)**	**Depression (HADS)**	**Disease duration**
**Healthy volunteers**	28 (13 saline; 15 naloxone)	39.9 ± 12.5	25 female; 3 male	157.1 lb ± 33.9	4.93 ± 3.11	1.93 ± 1.73	*NA*
**Chronic pain (FM) patients**	24 (11 saline; 13 naloxone)	43.7 ± 13.3	23 female; 1 male	160.3 lb ± 34.4	8.35 ± 4.55	4.74 ± 2.99	10.3 ± 7.4 years since diagnosis; 11.2 ± 6.8 years since reported symptom onset; mean FIQ score 43.7 ± 19.7

Chronic pain patients were included if they had had chronic widespread pain for at least 1 year prior to study participation with an average daily intensity of at least 4 of 10. FM diagnosis was confirmed through medical records. All participants were excluded for smoking (>10 cigarettes per week), excessive alcohol use (>7 drinks/week or 5 drinks in 1 setting), recreational drug use, pregnancy or breastfeeding, major medical or psychiatric conditions (past or present), recent use of opioids, and MRI contraindications. Non-opioid medications used to treat FM at the standard doses in the community were permitted. Healthy controls were excluded if they had taken any pain medication other than an over the counter NSAID or acetaminophen within the last 1 month or for >1 month on a continual basis within the last 6 months.

Chronic pain patients were included if they had had widespread chronic pain for at least 1 year prior to study participation with an average daily intensity at least 4 of 10. FM diagnosis was confirmed through medical records. All participants were excluded for smoking (>10 cigarettes per week), excessive alcohol use (>7 drinks/week or 5 drinks in 1 setting), recreational drug use, pregnancy, or breastfeeding, major medical or psychiatric conditions (past or present), recent use of opioids, and MRI contraindications. Non-opioid medications used to treat FM at the standard doses in the community were permitted. Healthy controls were excluded if they had taken any pain medication other than an over the counter NSAID or acetaminophen within the last 1 month or for >1 month on a continual basis within the last 6 months.

### Procedure

**Figure 1.**
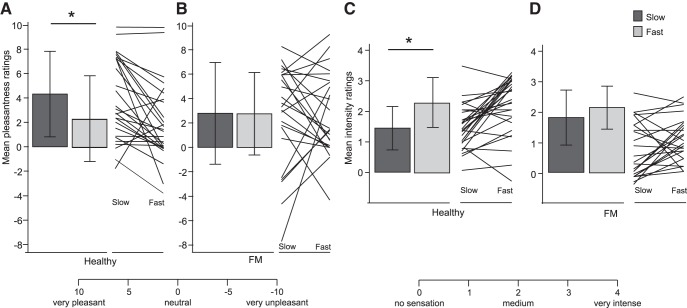
Pleasantness and intensity of brushing in healthy participants and chronic pain patients. Healthy participants and FM patients rated the pleasantness (***A***, ***B***) and intensity (***C***, ***D***) of slow (CT-optimal) and fast brushing of the left forearm on the corresponding VAS scales. Mean ratings at baseline (before any drug administration) are displayed; error bars show SD. *Two-tailed Tukey test, *p* < 0.05. Lines display individual participant data. There was a significant main effect of brushing speed (slow vs fast) on pleasantness ratings (*F*_(1,50)_ = 3.56, one-tailed *p* = 0.032 ^a^; without males: *F*_(1,46)_ = 3.76, one-tailed *p* = 0.027) but no main effect of group (healthy vs FM; *F*_(1,50)_ = 0.41, one-tailed, *p* = 0.26 ^b^; without males: *F*_(1,46)_ = 0.32, one-tailed, *p* = 0.26). There was a significant interaction between brushing speed and group (*F*_(1,50)_ = 3.32, one-tailed, *p* = 0.037^c^; Cohen’s *d* = 0.51; without males: *F*_(1,46)_ = 3.14, one-tailed, *p* = 0.04; ***A*** shows mean slow–fast ratings). *Post hoc* Tukey tests showed that healthy participants rated slow brushing as significantly more pleasant than fast brushing (Tukey, *p* = 0.042), whereas FM participants did not (Tukey, *p* = 1.00; ***A*** shows mean slow–fast ratings). Age did not affect ratings of brushing pleasantness or interact with speed in healthy participants (*F*_(1,26)_ = 0.03, *p* = 0.86^d^; *F*_(1,26)_ = 0.09, *p* = 0.76^e^) or in FM patients (*F*_(1,22)_ = 0.56, *p* = 0.46^f^; *F*_(1,22)_ = 3.08, *p* = 0.09^g^). When depression and anxiety were added to the model, depression significantly predicted pleasantness ratings (*F*_(1,46)_ =4.28, *p* = 0.04); anxiety did not (*F*_(1,46)_ = 0.42, *p* = 0.52). Including these ratings in the model strengthened the group by speed interaction (*F*_(1, 48)_ = 4.42, two-tailed, *p* = 0.041). There was a significant main effect of speed of brushing (slow vs fast) on intensity ratings (*F*_(1,50)_ = 4.26, *p* < 0.001^h^; without males: *F*_(1,46)_ = 20.0, *p* < 0.001) but no main effect of group (healthy vs FM; *F*_(1,50)_ = 0.32, one-tailed, *p* = 0.58 ^i^; without males: *F*_(1,46)_ = 0.19, two-tailed *p* = 0.67). There was a significant interaction between brushing speed and participant group (*F*_(1,50)_ = 4.26, *p* = 0.044 ^j^; Cohen’s *d* = 0.57; without males: *F*_(1,46)_ = 4.42, *p* = 0.041^,^). *Post hoc* Tukey tests showed that healthy participants rated fast brushing as more intense than slow brushing (Tukey *p* < 0.001), whereas FM participants did not (Tukey *p* = 0.24; ***B*** shows mean slow–fast ratings). Age did not affect ratings of brushing intensity or interact with speed in either healthy participants (*F*_(1,26)_ = 1.09, *p* = 0.31^k^; *F*_(1,26)_ = 0.11, *p* = 0.75^l^) or FM patients (*F*_(1,22)_ = 0.01, *p* = 0.93^m^; *F*_(1,22)_ = 0.05, *p* = 0.83^n^). Anxiety significantly predicted pleasantness ratings (*F*_(1,46)_ = 6.66, *p* = 0.01); depression did not (*F*_(1,46)_ = 1.34, *p* = 0.25). Including these ratings in the model weakened the group by speed interaction (*F*_(1,48)_ = 3.67, two-tailed *p* = 0.061).

As part of a larger study investigating placebo analgesia in patients with chronic pain, healthy participants and FM patients received slow and fast brushing stimuli on the left forearm, a region with significant CT fiber innervation (Vallbo et al., 1999), both before and after double-blinded intravenous administration of naloxone or saline. Participants received three trials of slow (3 cm s^−1^) brushing and three trials of fast (30 cm s^−1^) brushing (10-cm-long brushing strokes, 6 s per trial, 3 repetitions of slow brushing and 30 repetitions of fast brushing) in alternating order, beginning with slow brushing. Brushing was performed with a 2-inch-diameter watercolor brush (Mop and Wash Hake white goat-hair brush, force applied ∼0.7 N). Subjects rated both touch intensity and pleasantness/unpleasantness on 17 cm visual analog scales (VAS). Anchors for the intensity scale were no sensation (0) and very intense (4). A 17 cm VAS was also used for the affective scale, but in order to emphasize the difference between intensity and affective ratings, numeric anchors were 10 and −10, with the corresponding words of very pleasant and very unpleasant (Fig. 1); similar scales have been successfully used in previous studies (Triscoli et al., 2013; Croy et al., 2014; Jönsson et al., 2015). Participants marked a line on each scale to indicate their response. Participants were introduced to the brushing scale during a previous test session. Brushing was conducted by a male experimenter with the subject in an upright seated position (5 healthy subjects were brushed by a female experimenter). The experimenters had practiced the brushing procedure to ensure consistent stimulation force and velocity. There was no apparent effect of experimenter on the rating data.

Participants were randomly assigned (before the study began) to receive saline or naloxone in a double-blinded and counterbalanced manner. A maximum dose of 10 mg naloxone, a dose used clinically to reverse the effects of opiates, was administered to one-half of the subjects during an fMRI scan conducted for a separate part of the larger study. To achieve a constant plasma level throughout the MRI phase, a bolus dose of naloxone (0.05 mg/kg bodyweight; generic) or saline was first administered via an intravenous line, followed by an intravenous infusion dose of 0.08 mg/kg/h naloxone (diluted in 250 ml of saline) or an infusion of saline, starting immediately after the bolus injection and continuing for ∼40 mins. Participants were asked to guess which drug they had received and were not better than chance. The brushing task was conducted before the MRI scan (before drug infusion) and again ∼10 min after completion of the infusion and concurrent scan. The half-life of naloxone is 30–80 min with an average of 64 ± 12 min (McEvoy, 2012).

The unrelated fMRI study involved the rating of painful heat stimuli. Participants received two blocks of painful heat stimuli, one before and one during drug infusion. A topical placebo manipulation to decrease pain on a small area on the leg was administered. The control spot on the leg was not affected by placebo, so we believe that our arm-brushing task was similarly unaffected. Further, the placebo analgesia was small and the response of patients and controls did not differ (data to be reported elsewhere). Most patients were free of clinical pain during testing (20 of 24 subjects pain-free before drug and 17 of 24 pain-free after drug). Ongoing clinical pain scores were on average 0.69 ± 0.17 pre-drug and 1.33 ± 2.46 post-drug (paired *t* test, *p* = 0.09; 0–10 scale). The average level of discomfort in patients was also minimal, both pre-drug (0.71 ± 1.57) and post-drug (0.98 ± 2.14; paired *t* test *p* = 0.34, 0–10 scale), with 19 of 24 patients reporting no discomfort at all.

### Data analysis

Participants’ VAS ratings were measured independently with a ruler by two experimenters blind to drug condition and patient group. Ratings were averaged across trials separately for slow and fast brushing intensity and pleasantness. All analyses were conducted in JMP (SAS Institute). A two-factor ANOVA was conducted to test the effect of speed (slow vs fast) and group (healthy vs chronic pain) on baseline pleasantness ratings and separately on baseline intensity ratings. Significant effects were followed up with *post hoc* Tukey tests. Next, for each group, we conducted a two-factor ANOVA to test the effect of speed (slow vs fast) and drug (naloxone or saline) on pleasantness rating change scores (from before to after drug administration). We also investigated the effect of drug administration on average pleasantness ratings within the naloxone and saline conditions separately. The same analyses were conducted for ratings of intensity. Finally, we analyzed the effects of group, drug, and pre-post drug change in slow–fast preference (all main effects and interactions) on change in overall touch pleasantness. Slow–fast preference was calculated as each subject’s average slow brushing pleasantness rating minus average fast brushing pleasantness rating.

## Results

### Healthy subjects, but not chronic pain patients, rated fast and slow brushing differently

Healthy participants rated slow brushing of the skin as more pleasant than fast brushing, but less intense (Fig. 1a,*c*; repeated-measures ANOVA and *post hoc* Tukey’s; *p* values <0.05). In contrast to healthy subjects, chronic pain patients did not rate either the pleasantness or intensity of slow and fast brushing differentially (Fig. 1b,*d*; *p* values >0.2). Whereas pain patients differed from healthy subjects in the differential perception of slow and fast brushing, pain patients did not differ from healthy subjects in their average ratings of intensity or pleasantness (slow and fast brushing combined; main effects of group; *p* values >0.2). There was no effect of age on either intensity or pleasantness ratings for either healthy subjects or pain patients (*p* values >0.3) when included as a continuous covariate in the ANOVA. However, higher depression scores significantly predicted higher pleasantness ratings, whereas higher anxiety scores significantly predicted higher intensity ratings.

### Naloxone altered touch perception differently in chronic pain patients and healthy subjects

**Figure 2.**
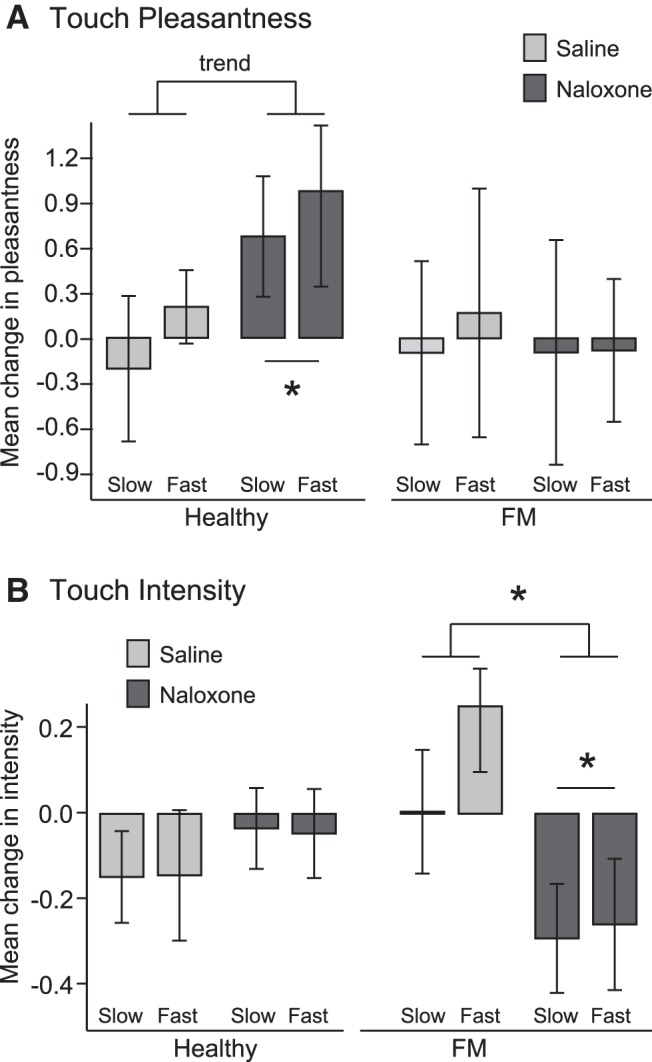
Effect of naloxone on pleasantness and intensity ratings of gentle touch in healthy participants and chronic pain patients. Healthy and FM participants rated the pleasantness (***A***) and intensity (***B***) of slow (CT-optimal) and fast brushing on the left forearm on a VAS scale before and after administration of naloxone or saline. Change scores (post – pre drug) in ratings of slow and fast brushing are displayed; error bars show SEM. ***A***, *One-tailed, *p* < 0.05; trend, one-tailed, *p* = 0.058. ***B***, *Two-tailed, *p* < 0.05. For healthy subjects there was no effect of brushing speed on change in pleasantness scores (*F*_(1,26)_ = 0.64, *p* = 0.43^°^; without males: *F*_(1,23)_ = 0.75, *p* = 0.40) and no interaction of speed and drug (*F*_(1,26)_ = 0.64, *p* = 0.90^p^; without males: *F*_(1,23)_ = 0.07, *p* = 0.80). However, there was a marginal effect of drug (*F*_(1,26)_ = 2.67, one-tailed *p* = 0.058^q^; *d* = 0.61; without males: *F*_(1,23)_ = 1.77, one-tailed *p* = 0.10). Within the naloxone group, naloxone caused a marginal increase in average pleasantness ratings (*t*_(14)_ = 1.98, two-tailed *p* = 0.067^r^; ***A***). There was no effect of saline in the saline group (*t*_(12)_ = 0.00, two-tailed *p* = 0.99^s^). There was no effect of speed (*F*_(1,26)_ = 0.002, *p* = 0.97^t^; without males: *F*_(1,23)_ = 0.32, *p* = 0.58), drug (*F*_(1,26)_ = 0.65, *p* = 0.43^u^; without males: *F*_(1,23)_ = 0.34, *p* = 0.57), or interaction of speed and drug on ratings of intensity (*F*_(1,26)_ = 0.01, *p* = 0.94^v^; without males: *F*_(1,23)_ = 0.10, *p* = 0.75; ***B***). For FM patients there was no effect of brushing speed (*F*_(1,22)_ = 0.05, *p* = 0.83^w^; without males: *F*_(1,21)_ = 0.02, *p* = 0.90) or drug (*F*_(1,22)_ = 0.03, *p* = 0.87^x^; without males: *F*_(1,21)_ = 0.01, *p* = 0.94) on change in pleasantness scores and no interaction of speed and drug (*F*_(1,22)_ = 0.04, *p* = 0.84^y^; without males: *F*_(1,21)_ = 0.08, *p* = 0.79). There was no effect of brushing speed (*F*_(1,22)_ = 1.146, *p* = 0.24^z^; without males: *F*_(1,21)_ = 1.60, *p* = 0.22) or interaction between speed and drug (*F*_(1,22)_ = 0.86, *p* = 0.36^aa^; without males: *F*_(1,21)_ = 0.64, *p* = 0.43) on change in intensity scores, but there was an effect of drug on intensity scores (*F*_(1,22)_ = 5.58, *p* = 0.027^ab^; *d* = 0.97; without males: *F*_(1,21)_ = 5.49, *p* = 0.029). Naloxone decreased FM participants’ ratings of intensity (*t*_(12)_ = 2.27, *p* = 0.043^ac^).

When we compared changes in pleasantness and intensity ratings from before to after naloxone or saline administration, we found that naloxone altered pleasantness ratings in the healthy subjects and altered intensity ratings in the chronic pain patients. Figure 2 shows that healthy subjects who received naloxone had a marginally significant increase in their ratings of pleasantness (Fig. 2a), but no effect on ratings of intensity (Fig. 2b). Ratings of slow and fast brushing pleasantness were not differentially affected. In contrast, chronic pain patients who received naloxone showed no effect on pleasantness (Fig. 2a) but a significant decrease in ratings of stimulus intensity (Fig. 2b). Again, ratings of slow and fast brushing intensity were not differentially affected. Saline did not alter ratings in either the healthy subjects or the pain patients (Fig. 2). FIQ score was unrelated to the naloxone-induced decrease in intensity perception in pain patients (*F*_(1,9)_ = 0.48, *p* = 0.51^ad^ ; Table 3). Although there was substantial individual variability between individuals in brushing ratings and change scores, no brushing rating differences were found at baseline between participants subsequently randomized to receive naloxone versus saline (Table 2 shows baseline means and statistics). This suggests that the effect of naloxone can safely be interpreted as an effect of naloxone and not attributed to chance baseline variation between subjects.

**Table 2.** Baseline ratings

	**Slow pleasantness**	**Fast pleasantness**	**Slow intensity**	**Fast intensity**
**Healthy volunteers**					
Naloxone Saline	4.63 ± 3.73 4.05 ± 3.42 *t*_(26)_ = 0.43, *p* = 0.67	3.31 ± 3.70 1.42 ± 3.21 *t*_(26)_ = 1.45, *p* = 0.16	1.34 ± 0.62 1.53 ± 0.79 *t*_(26)_ = 0.72, *p* = 0.48	2.13 ± 0.89 2.42 ± 0.75 *t*_(26)_ = 0.93, *p* = 0.36
**Chronic pain (FM) patients**					
Naloxone Saline	2.30 ± 3.11 3.19 ± 5.00 *t*_(22)_ = 0.51, *p* = 0.62	1.80 ± 3.85 3.54 ± 2.85 *t*_(22)_ = 1.27, *p* = 0.22	1.50 ± 0.75 2.05 ± 0.97 *t*_(22)_ = 1.50, *p* = 0.15	2.21 ± 0.60 2.08 ± 0.80 *t*_(22)_ = 0.46, *p* = 0.65

Healthy participants and FM patients rated the pleasantness and intensity of slow (CT-optimal) and fast brushing of the left forearm on the corresponding VAS scales. Mean ratings ± SD at baseline (before any drug administration) are displayed for slow and fast brushing for the naloxone and saline groups for healthy participants and FM patients. The *t* tests show that before drug infusion, there were no significant differences in ratings between individuals who subsequently received naloxone versus saline.

### Naloxone affected the relationship between overall pleasantness and slow–fast preference

**Figure 3.**
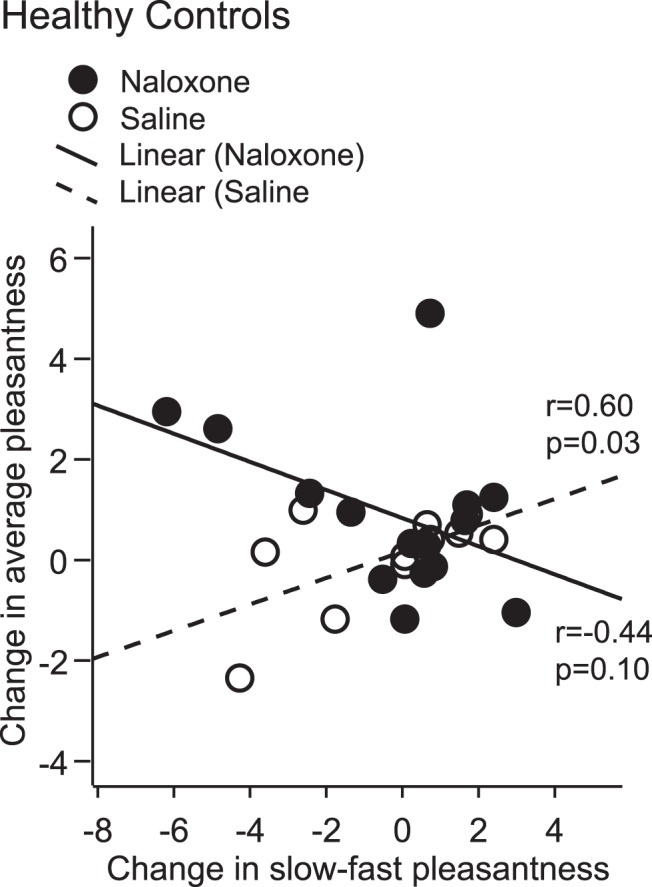
Effect of naloxone on change in touch pleasantness and preference for slow brushing. Healthy participants showed an effect of drug (naloxone versus saline) on the relationship between change in overall touch pleasantness and change in slow–fast preference (*F*_(1,24)_ = 6.55, *p* = 0.02^ae^; without males: *F*_(1,21)_ = 6.65, *p* = 0.02). Changes in overall pleasantness and changes in slow/fast preference were positively correlated under saline but negatively correlated (trend) under naloxone. Chronic pain patients did not show an effect of drug on the relationship between changes in overall intensity and changes in slow/fast intensity difference (not pictured; *F*_(1,20)_ = 0.06, *p* = 0.81^af^; without males: *F*_(1,19)_ = 0.08, *p* = 0.78).

In healthy participants who received saline, changes in touch pleasantness and changes in preference for slow brushing were positively correlated. Under naloxone, this correlation was abolished and a trend toward a negative correlation was found (Fig. 3). Chronic pain patients did not show differences between naloxone and saline in the relationship between changes in overall intensity and changes in slow/fast intensity difference.

## Discussion

In the current study, chronic pain patients with FM exhibited a blunted preference for CT-related touch pleasantness and touch intensity, compared to healthy matched participants. In addition, we demonstrated for the first time that opioid-blockade by naloxone altered touch perception, and did so differently for chronic pain patients than for healthy individuals. These findings suggest that opioids contribute to affective touch perception, and suggest abnormalities in the role of opioids in touch processing in patients with chronic pain.

### Chronic pain patients showed a blunted perception of CT-related touch intensity and pleasantness

In the current study, we replicated previous studies (Loken et al., 2009) showing that healthy adults find slow (CT-optimal) touch more pleasant than fast touch. As predicted, we found that chronic pain patients with FM have a reduced slow touch preference; indeed, on average, patients did not show any speed preference at all. We also observed that whereas healthy participants found fast brushing significantly more intense than slow brushing, FM patients did not; the rating distinction seen in healthy individuals was nearly halved in FM patients. The ratings of healthy and FM subjects differed by ∼10% on each rating scale, suggesting significant abnormalities in touch processing in chronic pain patients with FM. In comparison, clinical doses of morphine decrease pain by ∼30% on average (Kalso et al., 2004). The effect size for our rating changes are *d* = 0.51 for pleasantness and *d* = 0.57 for intensity, medium effect sizes by conventional criteria. In contrast, the mean effect size for placebo analgesia, a popular and meaningful topic of research, is *d* = 0.15 (Vase et al., 2002).

We do not believe these differences in touch perception are related to pain. Although FM patients do have tender points, light brush allodynia is not a typical feature of FM; in fact, “dry brushing” is a popular holistic treatment used by a number of FM patients. We do not have any indication that our light brushing of the skin caused pain in the FM patients in our study; indeed, average ratings of the unpleasantness/pleasantness of the brushing did not differ between healthy participants and FM patients. Similarly, although there is some evidence for lessened overall experience of pleasure in FM patients, such as reduced pleasantness ratings for pleasant odors (Schweinhardt et al., 2008), the lack of overall differences in touch pleasantness suggest similar levels of pleasure in FM patients. Instead, although gentle brushing stimulates both CT and A-beta fibers, the lack of preference for slow brushing suggests a particular difference in FM in processing of CT fibers, which are more strongly activated by slow (CT-optimized) speeds of brushing than by fast brushing. Intensity ratings are likely affected by both fiber types and thus less readily linked to CT fiber processing. Differences in brushing ratings were also predicted by depression and anxiety scores: higher depression scores predicted higher pleasantness ratings, while higher anxiety scores predicted higher intensity ratings. The effect of mood ratings did not remove the effect of patient group, however, suggesting that differences in FM touch perception are not mediated by mood.

Differences in CT touch processing in FM may be central or peripheral in origin. If opioid transmission underlies the appreciation of CT-optimal slow touch as we hypothesize, then degradation of central opioidergic transmission in chronic pain patients may explain why patients did not find CT-related brushing more pleasant. Indeed, there is evidence for an altered opioidergic system in FM. Harris et al. (2007) showed decreased central µ-opioid availability (expressed as decreased binding potential) using PET in 17 female FM patients compared with 17 age-matched healthy controls in several brain regions, including the nucleus accumbens, amygdala, and dorsal anterior cingulate, and some of these regional decreases were associated with greater clinical pain in the FM patients. Reduced opioid receptor binding potential within the CNS has also been shown in other chronic pain states including rheumatoid arthritis (Jones et al., 1994), neuropathic pain (Jones et al., 2004; Willoch et al., 2004; Maarrawi et al., 2007), and complex regional pain syndrome (Klega et al., 2010), though on occasion increases in brain opioid receptor availability have also been observed [eg, in CRPS (Klega et al., 2010) and back pain (Martikainen et al., 2013)]. Peripheral pathology is another possible source of abnormalities in CT processing in FM. Indeed, several studies have found individuals with FM to show small fiber pathology (Oaklander et al., 2013; Doppler et al., 2015).

### Naloxone increased the pleasantness of touch in healthy individuals

We directly tested the involvement of endogenous opioids in the perceived pleasantness of touch in FM patients and healthy controls. As predicted, we found that μ-opioid blockade by naloxone altered touch pleasantness in healthy participants. Touch pleasantness was increased by ∼10%, consistent with the majority of primate studies that report increased grooming (liking and wanting of brushing have been found to covary in previous studies; Triscoli et al., 2014). The effect in monkeys has been larger; Martel et al. (1995) found that mature female monkeys made 50% more solicitations and received 50% more grooming after naloxone. However, this and similar studies used doses of naloxone ∼0.5 mg/kg, ∼10 times higher than the current study. The magnitude of our finding is similar to the effect of naloxone on pain ratings (∼10%; Schull et al 1981) and the effect of a (much higher) dose of naloxone on mood ratings (also ∼10%; Cohen et al, 1983).

Contrary to our original hypothesis, naloxone did not show a differential effect on the pleasantness of slow versus fast touch. However, because slow and fast brushing both activate CT afferents (Loken et al., 2009), any differential effect might have been too weak to detect. These results suggest a role for endogenous opioids in the pleasantness of CT-related social touch, through either mediation or moderation of touch pleasantness representations. Indeed, the area most closely tied to the pleasantness of gentle touch in humans is the pgACC (Case et al., submitted; Lindgren et al., 2012), and the ACC has one of the highest densities of opioid binding receptors in the CNS (Jones et al., 1991; Sadzot et al., 1991; Vogt et al., 1995).

Mood may have played a role in the effect of naloxone on touch pleasantness. Panksepp’s Brain Opioid Theory of Social Attachment (BOTSA; Panksepp et al., 1978) proposes that social isolation leads to distress mediated by opioid withdrawal and negative affect, while social contact leads to positive emotions mediated by release of endogenous opioids. Building on BOTSA, (Loseth et al., 2014) have proposed the State-dependent µ-Opioid Modulation of Social Motivation (SOMSoM) which suggests that from an initial state of distress, opioid agonism provides comfort and thus reduces comfort seeking, whereas opioid blockade increases distress and provides stronger motivation for social comfort seeking (consistent with the monkey studies in which opioid blockade increases receipt of grooming). In contrast, from an initial state of comfort, opioid agonism enhances social exploration, whereas opioid blockade limits this behavior. In humans, numerous studies have also found that naloxone exerts a negative effect on mood that increases with dose (Grevert et al., 1983). Although we did not measure mood directly, our subjects were isolated in the MRI scanner and received painful heat stimulation during drug administration, which likely established an initial state of stress. Any interpretation of the effect of naloxone should include this likely state of stress. Baseline stress may have caused the opioid blockade to increase distress and heighten the social reward of affective touch. This interpretation suggests that opioids influence the motivational state that determines the reward and pleasantness of social touch.

We also found that in healthy individuals, changes in overall pleasantness and changes in slow–fast preference were positively correlated under saline but inversely correlated under naloxone. This relationship was not present in the pain patients, who lacked the overall effect of naloxone on pleasantness ratings. We speculate that naloxone might interfere with CT discrimination while simultaneously increasing the valuation of social touch overall. However, no overall effect of naloxone was found on CT discrimination, suggesting that any such effect was weak. A state of reduced opioid levels might diminish the distinction between fast and slow touch (based on decreased opioid neurotransmission involved in processing of CT signaling), but increase the overall valuation and liking of social touch.

### Naloxone altered the intensity of touch in chronic pain patients

In contrast to the effect observed in healthy controls, naloxone had no effect on touch pleasantness in chronic pain patients. Naloxone did, however, cause an unexpected decrease in patients’ ratings of brushing intensity (not differentiated by speed) that was not observed in healthy participants. Intensity ratings decreased by ∼5% on our rating scale but constituted a large effect size by conventional criteria (*d* = 0.97). Our dose of naloxone was low; a larger dose might show larger effects on patients’ ratings. It is not clear how opioids would become involved in touch intensity in chronic pain patients, but this effect may point to altered functions of the opioid system in FM patients, or to a change in function of CT fibers in chronic pain. Indeed, there is some evidence that in painful conditions, CT fibers may change their role from signaling pleasant touch to be involved in allodynia (Liljencrantz et al., 2013; Mahns and Nagi, 2013). Alternatively, changes in intensity perception could be related to observations in mice that opioids modulate the presynaptic activity of low threshold myelinated mechanosensitive afferents (Bardoni et al., 2014).

## Conclusion

In summary, we show for the first time that altered perception of touch intensity and pleasantness in chronic pain patients with proposed abnormalities of the opioid system. In addition, this is the first demonstration in humans that opioid blockade alters touch perception. In healthy individuals, opioid blockade marginally increased overall touch pleasantness (trend toward correlation with a decrease in CT slow touch preference), whereas in chronic pain patients with FM it significantly decreased overall touch intensity. These findings provide the first direct support in humans for the hypothesis that opioids have a role in CT-mediated affective qualities of touch. Our findings also provide further evidence for opioid abnormalities in patients with FM. The patients showed no preference for CT-optimal touch at baseline, and opioid blockade affected touch intensity rather than pleasantness, suggesting altered processing of CT input. These findings have significance in the understanding of human touch, as well as sensory processing in FM. More research is needed to determine whether abnormal touch perception and abnormal effects of opioids in fibromyalgia are related to the causes or consequences of chronic pain.

**Table 3.** Statistical table

	**Data structure**	**Type of test**	**Power**
**a**	Not normally distributed	ANOVA repeated measures, within factors	1
**b**	Not normally distributed	ANOVA repeated measures, between factors	0.92
**c**	Not normally distributed	ANOVA, repeated measures, within-between interaction	1
**d**	Not normally distributed	ANCOVA main effect	0.05
**e**	Not normally distributed	ANCOVA interaction	0.07
**f**	Not normally distributed	ANCOVA main effect	0.74
**g**	Not normally distributed	ANCOVA interaction	1
**h**	Not normally distributed	ANOVA repeated measures, within factors	1
**i**	Not normally distributed	ANOVA repeated measures, between factors	0.74
**j**	Not normally distributed	ANOVA, repeated measures, within-between interaction	1
**k**	Not normally distributed	ANCOVA main effect	1
**l**	Not normally distributed	ANCOVA interaction	0.09
**m**	Not normally distributed	ANCOVA main effect	0.05
**n**	Not normally distributed	ANCOVA interaction	0.06
**o**	Not normally distributed	ANOVA, repeated measures, within factors	1
**p**	Not normally distributed	ANOVA, repeated measures, within-between interaction	1
**q**	Not normally distributed	ANOVA, repeated measures, between factors	1
**r**	Not normally distributed	*t* test: one-sample	0.46
**s**	Not normally distributed	*t* test: one-sample	0.05
**t**	Not normally distributed	ANOVA, repeated measures, within factors	0.08
**u**	Not normally distributed	ANOVA, repeated measures, between factors	0.97
**v**	Not normally distributed	ANOVA, repeated measures, within-between interaction	0.05
**w**	Not normally distributed	ANOVA: repeated measures, within factors	0.06
**x**	Not normally distributed	ANOVA, repeated measures, between factors	0.05
**y**	Not normally distributed	ANOVA, repeated measures, within-between interaction	0.07
**z**	Not normally distributed	ANOVA: repeated measures, within factors	1
**aa**	Not normally distributed	ANOVA, repeated measures, within-between interaction	1
**ab**	Not normally distributed	ANOVA, repeated measures, between factors	1
**ac**	Not normally distributed	*t* test: one-sample	0.56
**ad**	Not normally distributed	Linear multiple regression	0.30
**ae**	Not normally distributed	ANOVA, repeated measures, within-between interaction	1
**af**	Not normally distributed	ANOVA, repeated measures, within-between interaction	0.09

## Synthesis

The decision was a result of the Reviewing Editor Mary Heinricher and the peer reviewers coming together and discussing their recommendations until a consensus was reached. A fact-based synthesis statement explaining their decision and outlining what is needed to prepare a revision is listed below. The following reviewers agreed to reveal their identity: Roger Fillingim

This study examined the role of endogenous opioids in the magnitude and pleasantness of sensations evoked by brushing in healthy individuals and patients with fibromyalgia. The findings are surprising in showing reduced pleasantness of slow touch in fibromyalgia, since this disorder is normally thought of as a "pain" syndrome. In addition, blocking endogenous opioids had different effects on touch perception in the healthy controls and fibromyalgia patients. Again, we normally think of opioids as modulating pain, so an effect on innocuous touch is surprising and interesting. It will be exciting to explore out the mechanisms underlying altered perception of light touch in these patients and where opioids come into the picture.
